# Low plasma vitamin D is associated with adverse colorectal cancer survival after surgical resection, independent of systemic inflammatory response

**DOI:** 10.1136/gutjnl-2018-317922

**Published:** 2019-04-25

**Authors:** P G Vaughan-Shaw, L Zgaga, L Y Ooi, E Theodoratou, M Timofeeva, V Svinti, M Walker, F O’Sullivan, A Ewing, S Johnston, F V N Din, H Campbell, S M Farrington, M G Dunlop

**Affiliations:** 1 Cancer Research UK Edinburgh Centre and MRC Human Genetics Unit, Institute of Genetics and Molecular Medicine, University of Edinburgh, Edinburgh, UK; 2 Department of Public Health and Primary Care, Trinity College Dublin, Dublin 24, Republic of Ireland; 3 Centre for Global Health Research, Usher Institute for Population Health Sciences and Informatics, University of Edinburgh, Edinburgh, UK; 4 Cancer Research UK Edinburgh Centre, Institute of Genetics and Molecular Medicine, University of Edinburgh, Edinburgh, UK; 5 Specialist Endocrine Laboratory, NHS Greater Glasgow and Clyde, Glasgow, UK

**Keywords:** colorectal cancer, cancer prevention, colorectal surgery, vitamin D receptor gene

## Abstract

**Objective:**

We assessed the effect of surgical resection of colorectal cancer (CRC) on perioperative plasma vitamin D (25OHD) and C-reactive protein (CRP) level. We investigated the relationship between circulating vitamin D level and CRC survival.

**Design:**

We sequentially sampled 92 patients undergoing CRC resection, and measured plasma 25OHD and CRP. For survival analyses, we assayed 25OHD and CRP in two temporally distinct CRC patient cohorts (n=2006, n=2100) and investigated the association between survival outcome, circulating vitamin D and systemic inflammatory response.

**Results:**

Serial sampling revealed a postoperative fall (mean 17.3 nmol/L; p=3.6e-9) in plasma 25OHD (nadir days 1–2). CRP peaked 3–5 days postoperatively (143.1 mg/L; p=1.4e-12), yet the postoperative fall in 25OHD was independent of CRP. In cohort analyses, 25OHD was lower in the 12 months following operation (mean=48.8 nmol/L) than preoperatively (54.8 nmol/L; p=1.2e-5) recovering after 24 months (52.2 nmol/L; p=0.002). Survival analysis in American Joint Committee on Cancer stages I–III demonstrated associations between 25OHD tertile and CRC mortality (HR=0.69; 95% CI 0.46 to 0.91) and all-cause mortality (HR=0.68; 95% CI 0.50 to 0.85), and was independent of CRP. We observed interaction effects between plasma 25OHD and rs11568820 genotype (functional *VDR* polymorphism) with a strong protective effect of higher 25OHD only in patients with GG genotype (HR=0.51; 95% CI 0.21 to 0.81). We developed an online tool for predicted survival (https://apps.igmm.ed.ac.uk/mortalityCalculator/) that incorporates 25OHD with clinically useful predictive performance (area under the curve 0.77).

**Conclusions:**

CRC surgery induces a fall in circulating 25OHD. Plasma 25OHD level is a prognostic biomarker with low 25OHD associated with poorer survival, particularly in those with rs11568820 GG genotype. A randomised trial of vitamin D supplementation after CRC surgery has compelling rationale.

**Significance of this study**

**What is already known on this subject?**

- Previous studies indicate that low circulating 25-hydroxyvitamin D (25OHD) is associated with poor colorectal cancer (CRC) survival yet sample timing and inflammatory response to cancer or surgery may confound these findings.

**What are the new findings?**

- We show that circulating 25OHD falls after CRC surgery independent of C-reactive protein (CRP), a measure of systemic inflammatory response.
- We show that the association between low vitamin D and adverse outcome in two large cohorts is independent of time of sample and CRP.

**How might it impact on clinical practice in the foreseeable future?**

- Vitamin D level may provide value to existing clinicopathological data in survival prediction and clinical decision-making.
- Vitamin D deficiency is a modifiable risk factor associated with survival outcome after CRC surgery.
- These data support a randomised trial of vitamin D after CRC surgery.

## Introduction

Observational studies implicate vitamin D deficiency in the aetiology and outcome of several common cancers. Available evidence suggests an inverse association between circulating 25-hydroxyvitamin D (25OHD) and colorectal cancer (CRC) risk and outcome.^1–4^ However, a causal relationship remains to be definitively established.^5^ Mendelian randomisation studies have failed to support a causal inference.^6–8^ However, one randomised controlled trial to date has shown a beneficial effect of vitamin D supplementation on CRC outcome.^1 9 10^ Absence of benefit may reflect inadvertent confounding or stratification, low statistical power, inclusion of study subjects with sufficient baseline levels, inadequate supplementation or insufficient follow-up.^11^ It is also possible that the association between CRC and vitamin D status is not causal, but rather a biomarker of ill health or inflammation.^9^ Furthermore, the inverse association could be due to reverse causality, with CRC, or its treatment, inducing lower vitamin D levels.^9 12–15^

Abdominal surgery is a major physiological insult, yet there are no studies of the effect of CRC surgery on circulating vitamin D. Published work demonstrates 25OHD (the best marker and storage form of vitamin D) decreases following orthopaedic, cardiac and gynaecological surgery.^14 16–20^ Several explanations are proposed to explain the observed changes, including circulatory fluid changes, that is, haemodilution^19 21^ and/or systemic inflammatory response (SIR) to surgery. However, there is incomplete understanding of the relationship between 25OHD and C-reactive protein (CRP, an established biomarker of inflammation).^20^

Previous work suggests that 25OHD is associated with survival outcome after a diagnosis of CRC.^22–28^ However, such a link may be confounded by the influence of surgery on 25OHD. Furthermore, there is evidence that the preoperative and postoperative inflammatory state itself is associated with survival.^29 30^ Circulating 25OHD has been shown to be associated with survival in patients with melanoma, independent of inflammatory response.^31^ However, there are no studies to date that have investigated the role of inflammation in the relationship between 25OHD and CRC survival.

We set out to investigate temporal variation in circulating 25OHD and CRP during the perioperative period by serial sampling of patients undergoing CRC resectional surgery. We explored the relationship between plasma CRP and 25OHD levels in a cohort of patients with CRC previously reported by us^22^ and conducted a replication study in a larger independent cohort. We assayed total circulating 25OHD CRP (biomarker of SIR) and genotyped individuals for a functional *VDR* single nucleotide polymorphism (SNP) (rs11568820) to investigate gene-environment effects. We then conducted multivariable survival analysis in patients with CRC undergoing resectional surgery with curative intent to determine the contribution of 25OHD and CRP to outcome.

## Methods

### Study population

Patients were from the Study of Colorectal Cancer in Scotland (SOCCS/SOCCS3) study, a population-based case–control study designed to identify genetic and environmental factors that have an impact on CRC risk and survival outcome.^32^ The research was subject to approvals from the National Research Ethics Committee and National Health Service management. All participants provided informed written consent. Clinical variables were collected from patient clinical records and pathology reports. All data were entered into an anonymised prospective study database and extracted for analysis.

To explore temporal variation in 25OHD and CRP in the perioperative period, we serially sampled 92 patients during the preoperative and immediate postoperative period. In the prospective observational study, we assayed plasma prospectively collected from two temporally distinct cohorts: c*ohort 1* (2001–2006) and *cohort 2* (2009–2016) for 25OHD and CRP levels.

We excluded patients with metastatic disease at diagnosis and conducted survival analysis in patients undergoing resectional surgery with curative intent (American Joint Committee on Cancer [AJCC] stages I–III). An updated survival analysis was conducted for *cohort 1*, including extended follow-up data and incorporating CRP level to explore whether the previously reported association between survival and plasma 25OHD^22^ was independent of SIR. We then set out to replicate the association between 25OHD and survival in a larger cohort of patients with CRC (*cohort 2*).

### Plasma vitamin D and CRP assay

In the *serial sampling study*, multiple samples were taken from each patient at preoperative assessment clinic intraoperatively and postoperatively (ward or outpatient clinic). Participants of *cohorts 1 and 2* were sampled once at recruitment which was at various time points during their cancer investigations or follow-up. Prior published data indicated a likely 25OHD drop of 30%–40%,^17 20^ yet SD of the drop is not known. A limited paired power calculation suggested that 90 patients would be required in the serial sampling study to demonstrate a 30% drop (80% power; alpha 0.05).

Plasma was extracted from blood sampled by venepuncture and submitted for liquid chromatography tandem mass spectrometry measurement of 25OHD at the Specialist Endocrine Laboratory (Glasgow Royal Infirmary) and for CRP assay at the NHS Lothian Biochemistry Laboratory. Blood leucocyte DNA was extracted by standard protocols and genotyped for rs11568820 using an Illumina Infinium array or DNA sequencing. Further details on assay, strict quality control and genotyping are provided in the online supplementary methods.

10.1136/gutjnl-2018-317922.supp1 Supplementary file 1

### Patient, tumour and treatment-related variables

We adjusted statistical models for patient-related factors previously established to influence 25OHD levels (age, sex, body mass index [BMI]) and AJCC stage. Survival data were collated from flagging research subjects in the Scottish national records system. Follow-up was determined by date of surgery, death or censor date (1 July 2017) for patients not known to be dead.

### Data analysis

For plasma 25OHD levels below the lower threshold of detection level, values were imputed in order to assign a value for inclusion in association analyses (online supplementary methods). May adjustment^33^ to account for seasonable variation in level is also described there. Statistical analysis was conducted in R,^34^ with univariate comparisons of vitamin D level performed using the Wilcoxon signed-rank test.

In the serial sampling study, we tested the association between sample time point and 25OHD using multivariable generalised linear mixed effects modelling. This model accounts for variability between patients (ie, assigned as a random effect). In cohorts 1 and 2, multivariable linear regression modelling was used for single measures of 25OHD to test for association between sample time point and 25OHD. Χ^2^ test was used to compare proportions of patients with deficient and sufficient levels of 25OHD at different time points. Survival analysis was performed using Cox proportional hazards models to calculate HRs, adjusting for other relevant factors including CRP and time from surgery to sample date. Main effects of the *VDR* polymorphism and its multiplicative interaction with vitamin D level on survival were assessed using a Cox proportional hazards model, providing a p value for interaction.

Individual cohort survival models excluding, and including, vitamin D level were compared using an analysis of variance likelihood ratio test with Harrell’s C statistic calculated in R (details in online supplementary methods).

## Results

### Serial sampling of plasma 25OHD in the perioperative period

**Figure 1:**
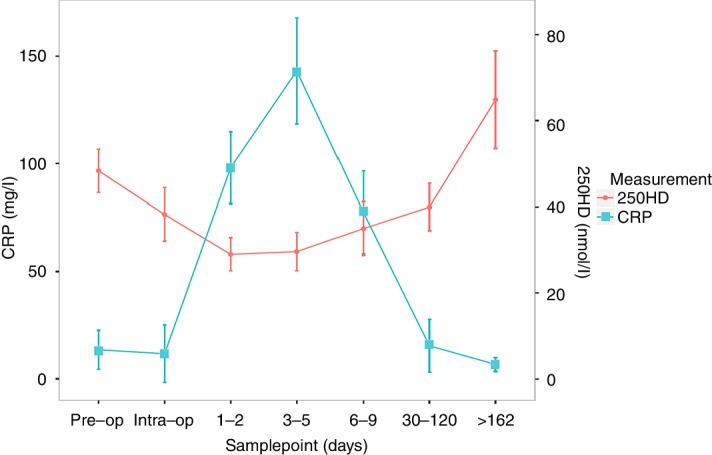
Perioperative 25OHD and CRP in patients undergoing colorectal cancer surgery. Unadjusted 25OHD charted with CRP. Sixty-two patients undergoing colorectal cancer surgery sampled at up to six perioperative time points. Mean 25OHD and CRP for each time point category given with error bars representing SD. 25OHD, 25-hydroxyvitamin D; CRP, C-reactive protein.

A total of 92 patients (table 1) underwent serial perioperative sampling at up to six time points. Mean 25OHD level reduced from 48.3 nmol/L preoperatively to 38.2 nmol/L intraoperatively (p=0.0004), then 29.0 nmol/L at 1–2 days following surgery (p=3.6e-9, figure 1, table 2). 25OHD began to recover within a week of surgery and increased to a level above the preoperative level at final sampling (64.9 nmol/L), a median of 225 days following surgery. Within-subject perioperative 25OHD levels were significantly correlated with preoperative levels (eg, day 1–2 level/preoperative level Spearman correlation R=0.62, p=8.1e-8, at all postoperative time points R≥0.62, p<0.0001) indicating that although 25OHD drops postoperatively, the rank order of 25OHD levels remained consistent.

**Table 1:** Clinical characteristics of included patients

	Serial sampling study	Cohort study Time point analysis		Cohort study Survival analysis	
		Cohort 1	Cohort 2	Cohort 1	Cohort 2
n	92	2006	2100	1687	1848
Sampling years	2012–2016	2001–2006	2009–2017	2001–2006	2009–2017
Age (years)	67.1 (64.4–69.9)	60.1 (60.6–61.6)	67.2 (66.7–67.7)	61.5 (61.0–62.0)	67.6 (67.1–68.1)
Gender (M)	51 (55%)	1137 (57%)	1172 (56%)	970 (57%)	1024 (55%)
BMI (kg/m^2^)	26.1 (24.8–27.5)	26.6 (26.4–26.8)	27.5 (27.3–27.7)	26.6 (26.4–26.8)	27.6 (27.4–27.9)
Cancer site					
Colon	62 (67%)	1153 (57%)	1313 (63%)	962 (57%)	1161 (63%)
Rectum	30 (33%)	829 (41%)	761 (36%)	714 (42%)	687 (37%)
NA	0	24 (1%)	56 (3%)	11 (1%)	0 (0%)
Cancer stage					
AJCC 1	27 (29%)	383 (19%)	415 (20%)	383 (22%)	415 (22%)
AJCC 2	31 (34%)	666 (33%)	692 (33%)	666 (39%)	692 (37%)
AJCC 3	21 (23%)	638 (32%)	741 (35%)	638 (38%)	741 (40%)
AJCC 4	7 (8%)	268 (13%)	167 (8%)	0†	0†
NA	6 (7%)	51 (3%)	85 (4%)	0	0
‘raw’ 25OHD (nmol/L)	NA*	29.3 (28.4–30.2)	46.5 (45.3–47.6)	29.1 (28.1–30.1)	46.0 (44.7–47.4)
May-adjusted 25OHD (nmol/L)	NA*	33.9 (33.0–34.8)	51.7 (50.5–52.8)	28.8 (27.82–29.7)	52.1 (50.9–53.4)
Tertile 1	NA*	<18.5	<37.32	<18.1	<38.0
Tertile 2	NA*	18.5–34.0	37.3–56.5	18.1–33.1	38.0–57.9
Tertile 3	NA*	>34.0	>56.46	>33.1	>57.9
Sample time point					
Preoperative (months)	NA*	0 (0%)	560 (27%)	0 (0%)	486 (26%)
0–12	NA*	1798 (90%)	549 (26%)	1517 (90%)	471 (26%)
12–24	NA*	180 (9%)	396 (19%)	147 (9%)	351 (19%)
>24	NA*	28 (1%)	595 (28%)	23 (1%)	540 (29%)
Season of sample					
Winter	NA*	489 (24%)	573 (27%)	408 (24%)	517 (28%)
Spring	NA*	524 (26%)	563 (27%)	449 (27%)	483 (26%)
Summer	NA*	465 (23%)	434 (21%)	392 (23%)	378 (20%)
Autumn	NA*	528 (26%)	530 (25%)	438 (26%)	470 (25%)
Follow-up (days)	NA*	NA	NA	4841	1318
Survival					
Alive	NA*	NA	NA	978 (58%)	1637 (89%)
Dead	NA*	NA	NA	709 (42%)	211 (11%)
CRC death	NA*	NA	NA	421 (25%)	138 (7%)

Age at time of surgery given. Pre-illness BMI reported where available. Mean values given for summary variables except follow-up (median censored follow-up given). ‘Raw’ 25OHD was not adjusted for seasonal variation, that is, not ‘May-adjusted’.

*Repeated measures.

†AJCC 4 excluded from survival analysis.

25OHD, 25-hydroxyvitamin D; AJCC, American Joint Committee on Cancer; BMI, body mass index; CRC, C-reactive protein; NA, not applicable.

**Table 2:** May-adjusted 25OHD level in serial sampling study

Time point	n	25OHD (nmol/L)	Difference (nmol/L)	P value*	P value†
Preoperative	92	48.3 (43.3–53.4)	Reference	Reference	Reference
Intraoperative	30	38.2 (31.8–44.7)	−12.8	0.0004	7.8e-5
1–2	62	29.0 (25.1–32.9)	−17.3	3.6e-9	5.5e-7
3–5	58	29.6 (25.2–34.1)	−17.2	4.4e-9	6.5e-5
6–9	45	35.0 (28.7–41.2)	−12.7	3.0e-6	0.002
30–120	58	40.0 (34.4–45.5)	−6.4	0.002	0.04
>162	40	64.9 (53.5–76.2)	18.3	3.1e-5	2.0e-8

Difference in 25OHD at each time point accounts for missing samples within paired comparisons. Preoperative samples were taken median 7.5 days before surgery (range 49 to 0 day). >162 day samples taken median 225 days postoperatively (range 163–315 days).

*P values given represent paired Wilcoxon sign-rank test results with preoperative level as reference. C-reactive protein (CRP) was assayed in 70 patients preoperatively. Of these, 30 then had repeat CRP assayed in intraoperative samples while 40 patients had CRP assayed at various postoperative time points.

†P values given represent results from linear mixed model including the fixed covariates age, gender, body mass index (BMI) and CRP, and patient ID included as a random effect.

25OHD, 25-hydroxyvitamin D.

To investigate whether the fall in 25OHD level was a manifestation of the inflammatory response to surgery, CRP was assayed in 70 of the 92 patients (insufficient plasma available for remainder). CRP significantly increased after surgery, peaking at 3–5 days (mean 143.1 mg/L vs preoperative mean 13.5 mg/L, p=1.4e-12; figure 1; online supplementary table 1). However, there was no correlation between 25OHD and CRP levels at any early postoperative time point (R^2^<0.015, p≥0.5) and the fall in 25OHD was not correlated with concurrent increase in CRP (R^2^<0.02, p≥0.38). Adjusting for the 3–5 days’ lag in CRP increase, compared with the fall in 25OHD at 1–2 days, similarly revealed no apparent correlation. Using generalised linear mixed effects modelling, we observed significantly lower 25OHD for up to 120 days after surgery, even after adjustment for CRP (online supplementary table 2). This strongly suggests that the fall in plasma vitamin D level is independent of postsurgical SIR.

10.1136/gutjnl-2018-317922.supp2 Supplementary file 2

### 25OHD level and survival outcome in CRC patient cohorts

**Figure 2:**
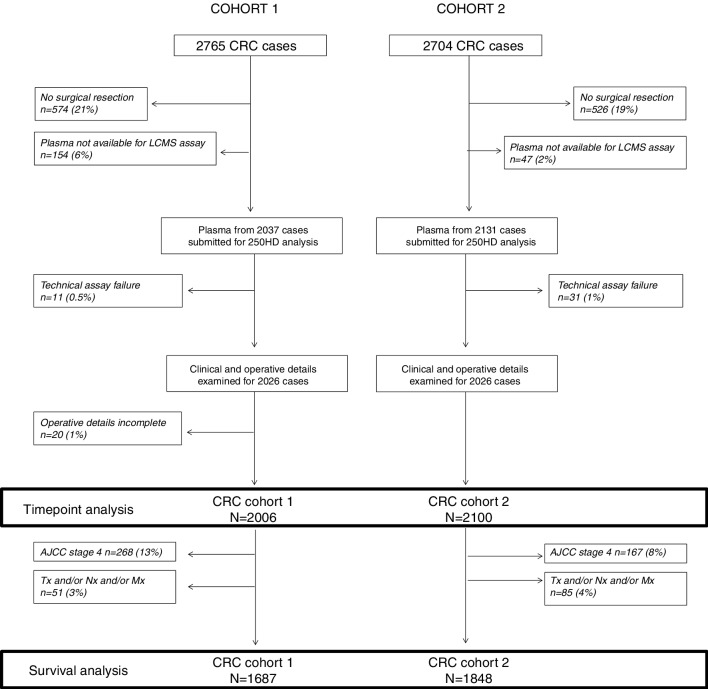
Flow chart of study participants. Patients were sampled from the Scottish colorectal cancer study from 2000 to 2006 (cohort 1) and from 2009 to 2017 (cohort 2, recruited up to 23 February 2017). *Tx and/or Nx and/or Mx indicates pathology report and AJCC stage not available, AJCC stage 4 indicates stage at time of surgery. Cohort 2 recruited and assayed up to 23 February2017. 25OHD, 25-hydroxyvitamin D; AJCC, American Joint Committee on Cancer; CRC, C-reactive protein; LCMS, liquid chromatography–mass spectrometry.

**Figure 3:**
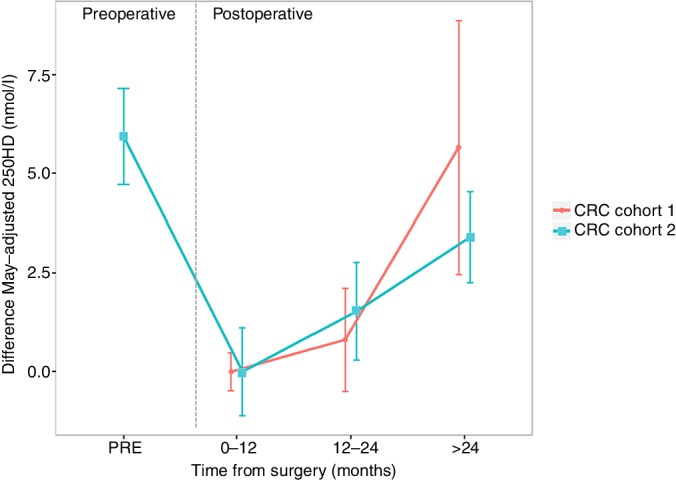
Difference in May-adjusted 25OHD levels by sample time point in cohorts. Mean May-adjusted 25OHD level at 0–12 months’ time point used as reference. 25OHD values scaled against reference mean and mean difference for each time point category given with error bars representing SEM. Cohort 1 (n=2006), cohort 2 (n=2100). 25OHD, 25-hydroxyvitamin D; CRC, C-reactive protein.

We assessed trends in 25OHD level over time from surgery with respect to survival (figure 2, table 1). We observed associations between 25OHD level and age, BMI and AJCC stage (online supplementary table 3). There was a trend towards increase in mean 25OHD over time from surgery in *cohort 1* (figure 3, online supplementary table 4). In *cohort 2,* mean 25OHD was significantly lower in samples taken 0–12 months following surgery (48.8 nmol/L) when compared with preoperative samples (54.8 nmol/L; p=1.2e-5) and late postoperative samples (>24 months, 52.2 nmol/L; p=0.002) (online supplementary table 4, figure 3), consistent with a pattern of postoperative fall and then recovery in 25OHD similar to that seen in the serial sampling study described above.

### 25OHD level, CRP and survival following CRC surgery

Plasma CRP was assayed in parallel with 25OHD in samples obtained from 1798 patients with CRC (404 preoperative cases from *cohort 2*, 1273 within 12 months of surgery and 121 cases >12 months from surgery from *cohort 1*). 25OHD and CRP were weakly correlated at all time points (preoperative samples R=−0.12, p=0.02; 0–12 months R=−0.14, p=1.2e-6; >12 months R=−0.16, p=0.03), with no clear difference in the strength of the correlation between 25OHD and CRP between the sampling time point categories, suggesting that the early postsurgical inflammatory response does not influence the weak observed association between 25OHD and CRP.

Survival data were analysed for *cohort 1* and *cohort 2* patients who underwent surgical resection with curative intent with follow-up data available for analyses (table 1, figure 2).

*Cohort 1* patients (n=1687) had a median follow-up of 13.3 years, with 709 deaths (421 CRC-specific deaths). Some 1223 patients had contemporaneously sampled CRP (mean 3.52, median 98 days postoperative), with the majority <10 mg/L (92%). Survival analysis adjusting for CRP level showed that higher postoperative 25OHD levels were independently associated with lower CRC-specific mortality (p=0.007) and all-cause mortality (p=0.0002; 25OHD as continuous variable). Comparing patients with May-adjusted 25OHD levels in the highest versus the lowest tertile, the fully adjusted HR (including adjustment for CRP) was 0.66 (95% CI 0.49 to 0.89) for CRC-specific mortality and 0.65 (95% CI 0.51 to 0.81) (table 3) for all-cause mortality, indicating that postoperative 25OHD influences survival outcome after a diagnosis of CRC independent of sample time point and the inflammatory response.

**Table 3:** Unadjusted and multivariable adjusted HRs of death in *cohort 1* and *cohort 2* according to May-adjusted 25OHD tertile

Model	n	Cohort 1							
		Tertile 1 <18.1 nmol/L	Tertile 2 18.1–33.1 nmol/L			Tertile 3 >33.1 nmol/L			P_trend_
		HR	HR	95% CI	P value	HR	95% CI	P value	
*CRC death*									
Model 1*	1685	Ref	0.84	0.67 to 1.06	0.15	0.74	0.58 to 0.93	0.01	0.05
Model 2†	1486	Ref	0.85	0.67 to 1.09	0.20	0.71	0.55 to 0.92	0.01	0.03
Model 3‡	**1058**	**Ref**	**0.77**	**0.59 to 1.04**	**0.09**	**0.66**	**0.49 to 0.89**	**0.006**	**0.007**
*All death*									
Model 1*	1685	Ref	0.77	0.65 to 0.92	0.004	0.70	0.58 to 0.84	0.0001	**0.0006**
Model 2†	1486	Ref	0.77	0.64 to 0.92	0.005	0.69	0.56 to 0.84	0.0002	0.0004
Model 3‡	**1058**	**Ref**	**0.72**	**0.58 to 0.91**	**0.006**	**0.65**	**0.51 to 0.81**	**0.0002**	**0.0002**

	n	Cohort 2							
		Tertile 1 <38.0 nmol/L	Tertile 2 38.0–57.9 nmol/L				Tertile 3 >57.9 nmol/L		P_trend_
*CRC death*		HR	HR	95% CI	P value	HR	95% CI	P value	
Model 1*	1844	Ref	0.70	0.47 to 1.04	0.08	0.56	0.37 to 0.86	0.008	0.10
Model 2†	**1792**	**Ref**	**0.77**	**0.52 to 1.13**	**0.19**	**0.62**	**0.40 to 0.95**	**0.03**	**0.13**
*All death*									
Model1*	1844	Ref	0.76	0.55 to 1.04	0.09	0.58	0.41 to 0.82	0.002	0.04
Model 2†	**1792**	**Ref**	**0.84**	**0.61 to 1.15**	**0.27**	**0.63**	**0.44 to 0.89**	**0.01**	**0.08**

Final model is shown in bold.

*Model adjusted for age, sex and American Joint Committee on Cancer (AJCC) stage.

†Multivariable model additionally adjusted for body mass index, tumour site (colon/rectum), time between definitive treatment and sampling. Where data were missing, participants were excluded from adjusted model reflected in decreasing numbers of patients included in each sequential model.

‡Multivariable model additionally adjusted for C-reactive protein (CRP) which is not displayed for cohort 2 as only a small subset had CRP assayed. Trend—25OHD as a continuous variable. Adjustment of model 3 for dietary intake of vitamin D or vitamin D supplementation did not substantially alter observed HRs.

25OHD, 25-hydroxyvitamin D; CRC, colorectal cancer.

To further explore these findings, we sought to replicate the association between plasma 25OHD and survival. We conducted further analysis in *cohort 2* (n=1848, median follow-up 3.6 years, range 52 days to 15 years), in which 211 deaths had occurred (138 CRC specific). Comparing patients with May-adjusted 25OHD levels in the highest versus the lowest tertile, the fully adjusted HR was 0.62 (95% CI 0.40 to 0.95) for CRC-specific mortality and 0.63 (95% CI 0.44 to 0.89) for all-cause mortality, thereby further strengthening previous findings in *cohort 1* (table 3).

Sensitivity analyses indicated that 25OHD was most strongly associated with survival of those patients sampled preoperatively (HR=0.30 all-cause death, 95% CI 0.12 to 0.71, online supplementary table 5). In cohort 2 patients assayed for CRP (all sampled preoperatively), 25OHD was again independently associated with all-cause mortality (tertile 3 vs tertile 1 HR=0.38, 95% CI 0.15 to 0.98) (online supplementary table 6). Finally, we undertook a recursive approach with 25OHD as a categorical threshold variable, in which a 25OHD threshold of ~45–50 nmol/L appears to most strongly associate with survival when considering both effect size and significance (online supplementary table 7).

### Meta-analysis of survival outcome

**Figure 4:**
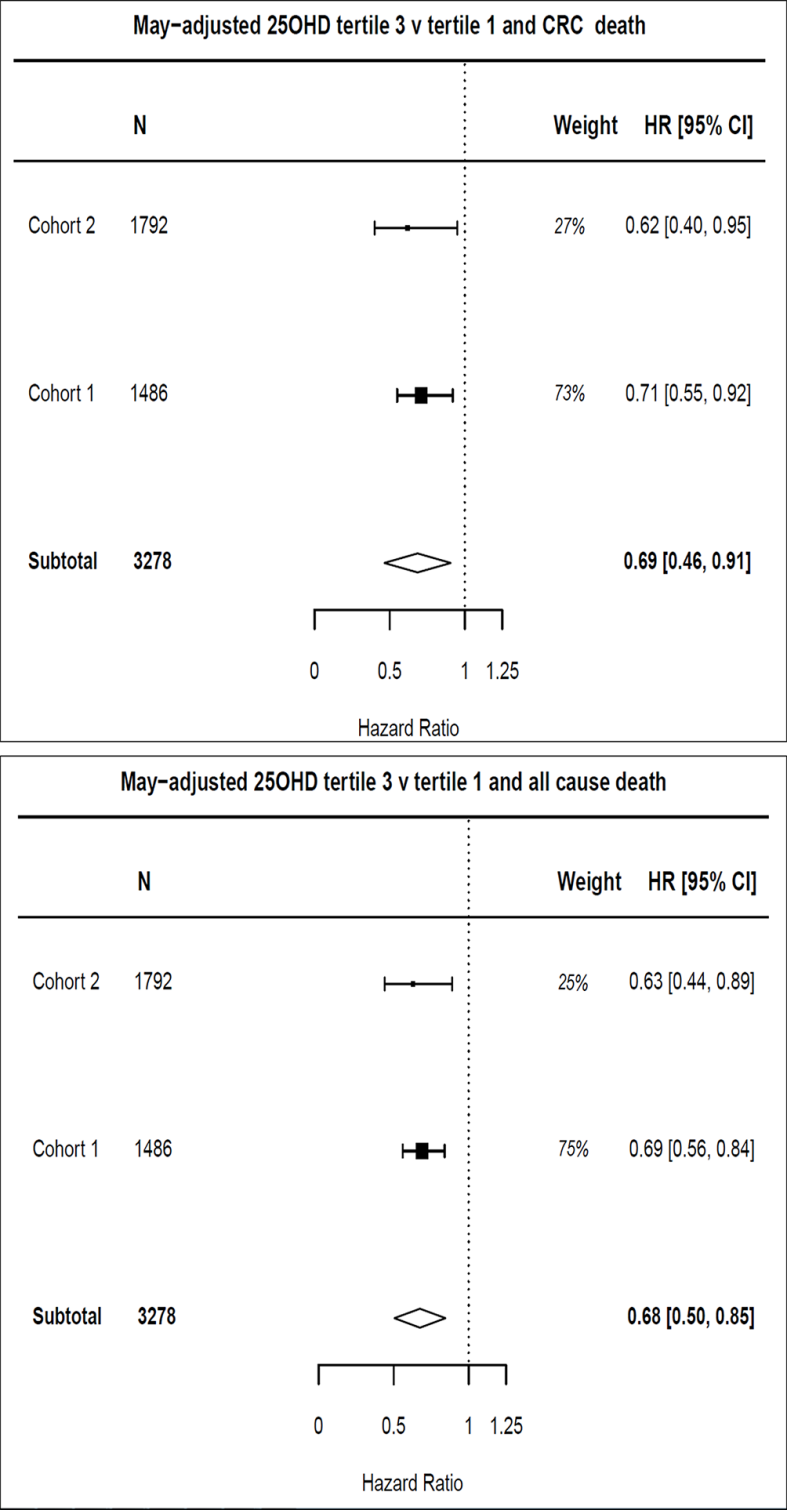
Meta-analysis of HR of 25OHD tertile 3 versus tertile 1 with colorectal cancer (CRC)-specific and all-cause mortality. Adjusted HRs used for meta-analysis, adjusted for age, sex, American Joint Committee on Cancer (AJCC) stage, body mass index, tumour site (colon/rectum) and time between definitive treatment and sampling. 25OHD, 25-hydroxyvitamin D; CRP, C-reactive protein.

We observed a cohort effect on mean vitamin D levels between cohorts 1 and 2 (mean 25OHD 28.8 and 52.1 nmol/L), despite rigorous internal controls in the assay laboratory. Despite this, meta-analysis demonstrates associations between May-adjusted 25OHD and both CRC-specific (tertile 3 vs tertile 1; HR=0.69, 95% CI 0.46 to 0.91) and overall survival (HR 0.68, 95% CI 0.50 to 0.85; figure 4) that are clinically meaningful. Meta-analysis of patients with contemporaneous CRP assay confirmed association with CRC-specific (HR=0.66, 95% CI 0.38 to 0.95) and all-cause mortality (HR=0.64 95% CI 0.42 to 0.85) independent of the SIR (online supplementary figure 1).

Although the higher 25OHD level in the more recent cohort may result from sample collection/storage/transportation factors, natural variability between/within study subjects, publicity about vitamin D leading to increased availability/ingestion of supplements and foodstuffs or differential sun exposure over time might have results in a true biological difference in level between the two cohorts. Therefore, we performed an adjusted survival analysis, comparing survival in *cohort 1* vs *cohort 2* irrespective of actual 25OHD level, which showed significantly improved cancer-specific and overall survival in *cohort 2* (HR=0.58, 95% CI 0.42 to 0.79 and HR=0.63, 95% CI 0.49 to 0.80, online supplementary table 8).

### Genotype at the rs11568820 VDR locus SNP and survival

**Figure 5:**
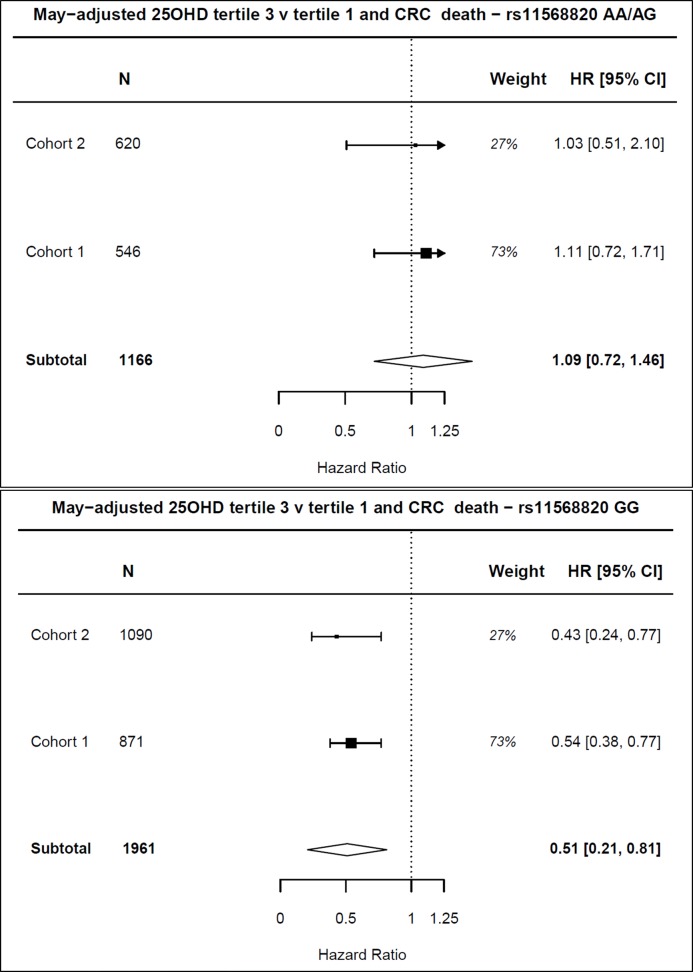
Meta-analysis of HR of 25OHD tertile 3 versus tertile 1 for colorectal cancer (CRC) mortality stratified by rs11568820 genotype. Adjusted HRs used for meta-analysis, adjusted for age, sex, American Joint Committee on Cancer (AJCC) stage, body mass index, tumour site (colon/rectum) and time between definitive treatment and sampling. 25OHD, 25-hydroxyvitamin D; CRP, C-reactive protein.

We confirmed previously reported evidence for gene–environment interaction effects on CRC-specific mortality between 25OHD level and genotype at a functional variant within the *VDR* gene sequence (rs11568820)^22^ in cohort 1, p=0.02. This remained significant after adjustment for CRP level (interaction p=0.003), and replicated this G×E in cohort 2 (interaction p=0.03) (online supplementary table 9). A meta-analysis of survival outcome stratified by genotype at rs11568820 supports the genetic interaction with vitamin D on survival. The association between 25OHD level and survival was particularly strong for the GG genotype (tertile 3 vs tertile 1 HR for CRC mortality in rs11568820 AA/AG HR=1.09; 95% CI 0.72 to 1.46; rs11568820 GG genotype HR=0.51; 95% CI 0.21 to 0.81, figure 5, online supplementary table 10). This observed G×E effect supports a causal association between vitamin D and survival outcome.

### Survival modelling and assessment of 25OHD clinical utility in prognosis

Comparison of the Cox proportional hazards models with/without 25OHD level found a modest improvement in the survival model including 25OHD level (likelihood ratio test p=2.9e-5, Harrell’s C statistic with/without 25OHD 0.67 and 0.66), indicating potential value of vitamin in survival prediction and clinical decision-making. Harrell’s C statistic was highest for preoperatively sampled patients (0.81) despite a relatively small sample size (n=451). To provide a clinically relevant model, we constructed a proof of concept logistic regression survival model based on survival at follow-up truncated 5 years (online supplementary table 11). Unadjusted 25OHD was modelled with sample month and sample time point (</>12 months following surgery), age, gender, cancer site and AJCC as covariates. In the model, 25OHD as a continuous variable was significantly associated with survival in both cohorts (*cohort 1* p=0.02; *cohort 2* p=0.015), with a significant and clinically relevant uplift in area under the curve (AUC) seen with inclusion of 25OHD in the model (*cohort 2* AUC excluding/including 25OHD level 0.74 and 0.77, respectively; online supplementary table 12). The *cohort 2* model for survival prediction at 5 years was published at https://apps.igmm.ed.ac.uk/mortalityCalculator/ with *cohort 2* chosen as it represents a more up-to-date population with greater spread of perioperative 25OHD sampling.

## Discussion

Serial sampling of patients undergoing resectional surgery for CRC demonstrates that surgery induces a fall in plasma vitamin D level, independent of CRP response. Plasma 25OHD levels sampled soon after surgery are lower when compared with those sampled later, consistent with a postoperative drop and longer term recovery in 25OHD. We found strong evidence of an association between lower vitamin D levels and adverse survival outcome in both cohorts and this association was independent of the SIR (as reflected by CRP). Furthermore, there is a strong genotype-specific effect of vitamin D, with survival association greatest in those with the *VDR* rs11568820 GG genotype. The findings have potential clinical relevance in survival prediction and clinical decision-making. While the mechanism underlying this observed association merits further study, this study supports the notion that vitamin D is a modifiable risk factor for survival outcome and provides compelling rationale for an intervention trial of supplementation after CRC surgery.

It is argued that the association between 25OHD and CRC risk/survival might reflect the influence of the SIR to CRC or its treatment on 25OHD.^9 15^ Indeed, CRP, a marker of inflammation, is also correlated with both CRC risk^35^ and survival.^29 36^ However, the current study provides evidence against CRP as a confounding variable in observational studies of 25OHD and CRC risk/outcome. First, the contribution to the variance of 25OHD by SIR is small. Second, we found no correlation between 25OHD drop following surgery and concurrent CRP increase while the postoperative drop in 25OHD was independent of changes in CRP, consistent with similar perioperative studies.^14 20^ Finally, 25OHD level was associated with both CRC and all-cause mortality, even after adjustment for CRP, supporting similar findings in patients with melanoma.^31^ Indeed, the relationship between postoperative 25OHD and survival appears stronger (effect size and greater statistical significance) after adjustment for CRP, despite smaller number of patients in adjusted analysis. Thus, after any variance in outcome explained by CRP is accounted for, the relationship between 25OHD and outcome becomes more apparent, providing further support for the independent effect of vitamin D on CRC survival.

Lower 25OHD levels are associated with higher CRC-specific and all-cause mortality even after adjustment for CRP and sample time from surgery. This survival association is consistent with numerous previous studies^2^ and indicates that plasma 25OHD level may be useful as a biomarker predicting survival outcome. We have developed a survival model including plasma 25OHD level with a clinically useful predictive performance (available at https://apps.igmm.ed.ac.uk/mortalityCalculator/). Furthermore, we have seen a strong genotype-specific effect of vitamin D, with survival association greatest in those with the *VDR* rs11568820 GG genotype. The combined HR in these patients is 0.51 (95% CI 0.21 to 0.81), indicating double the risk of CRC-specific death in patients with the lowest tertile of 25OHD. The rs11568820 variant is located in the *VDR* promoter region and directly inﬂuences transcriptional activity^37^ providing biological plausibility to this clinically relevant genotype-specific effect.

Limitations of this study include the possibility that unmeasured differences in demographics, genetic background or clinical factors might explain the observed differences in 25OHD. Second, 25OHD levels were different between the two cohorts. This may indicate real biological differences or could reflect differences in sample handling. The better survival in cohort 2 when compared with cohort 1 may reflect the improved vitamin D status in these patients due to the reasons stated above, yet may be partially or wholly explicable by advances in surgical, perioperative and oncological practice. To address these issues we have performed per-cohort analyses and meta-analysis. Finally, we cannot exclude the possibility that the perioperative drop in 25OHD is due to factors other than surgery itself (eg, anaesthetic, period of starvation).

Our data indicate that observed associations between 25OHD and CRC outcome are independent of the inflammatory response to CRC or its treatment and suggest that 25OHD level could be of utility in clinical decision-making, with the survival model provided here now meriting further validation. The observed gene interaction between 25OHD level and *VDR* genotype is consistent with a causal relationship between vitamin D and survival in patients with CRC. This study establishes that vitamin D deficiency is a modifiable risk factor associated with survival outcome from CRC. The findings provide compelling rationale for a randomised trial of vitamin D supplementation of deficient patients after CRC surgery with a defined endpoint of survival.
