# Supplementary material for: Low plasma vitamin D is associated with adverse colorectal cancer survival after surgical resection, independent of systemic inflammatory response
Source: Gut. 2019 Apr 25;69(1):103–11. doi: 10.1136/gutjnl-2018-317922 (PMC6943245; doi:10.1136/gutjnl-2018-317922)
Supplement: Supplementary file 1 [file gutjnl-2018-317922supp001.pdf]

Supplementary Methods

Plasma 25OHD assay

To minimise any potential analytical variation in plasma 25OHD measurement between the two different cohort study time periods, all samples were assayed in a single United Kingdom Accreditation Service (UKAS) accredited laboratory using a method traceable to National Standards of Science and Technology (NIST) standard reference material.[1] Total 25OHD (25OHD2 and 25OHD3) was measured by liquid chromatography tandem mass spectrometry. Plasma samples (2001-2006) were analysed using the Waters® ACQUITY™ TQD system. Subsequent samples were analysed using the Waters® Xevo® TQ-S instrument. Over the study time period, instrument migration was separately monitored as part of UKAS accreditation processes of the analysis laboratory. This comprises stringent external quality assurance performance measures to ensure perfect calibration. Additionally, to ensure comparability of assay calibration and acceptance criteria between the two CRC cohorts, 25OHD results were compared in 1000 external samples before/after instrument migration, confirming results were within analytical imprecision of the assay. Finally, blinded replicate plasma samples were submitted to the laboratory with 25OHD results exhibiting high intra-class correlation coefficient and low coefficient of variance (n=44 samples, correlation coefficient 0.97, 95%CI: 0.92-0.99; %CV 8%).

Plasma CRP assay

Samples were assayed for CRP, as a validated biomarker of systemic inflammatory response, in an NHS Biochemistry Laboratory serving our hospital. CRP was measured using the Abbott Architect C series clinical chemistry analyser to standard sensitivity protocol, with the range of output values 0.2-480 mg/l. CRP internal quality control was performed daily, with 3 control samples assayed twice per day. Target mean of the three control samples was 3.2, 8.3 and 27.5mg/l with actual observed mean over 6 months of 3.2, 8.4 and 27.8mg/l respectively from 5151 completed assays. Coefficients of variation were 3.42%, 2.34% and 2.05% respectively.

Genotype at SNPs relevant to vitamin D function or plasma level

Blood leukocyte DNA from patients in the cohorts 1 and 2 was genotyped by Illumina Infinium array or DNA sequencing for the functionally relevant *VDR* polymorphism rs11568820 with previous reported interaction effects on CRC survival.[2]

Patient, Tumour and Treatment-Related Variables

We adjusted for patient-related factors previously established to influence 25OHD levels (age, sex, body mass index (BMI)). [2-4] Tumour and treatment-related variables were collected to investigate putative effect on 25OHD, including American Joint Committee on Cancer (AJCC) stage; operative approach (open or laparoscopic) and perioperative intravenous fluid administration. Information on tumour site, multiplicity and clinico-pathologic staging were obtained from clinical records, along with preoperative imaging. By using collated pathology, imaging, and clinical data, tumour stage was mapped onto the AJCC staging system (AJCC stage I to IV). Perioperative intra-venous fluid volume was taken as the volume of intra-venous fluid administered on the day of operation (i.e. intra-operative and postoperative until midnight). Survival data was collated from follow-up through systematic search of the Scottish national records system. Deaths were ascertained through linkage to National Records of Scotland with primary cause of death (CRC, other cancer, other cause) assigned from death certificates. Follow-up was defined as the time between surgery date and death or censor date for patients who were not known to have died.

Data analysis

Where 25OHD was reported as below the lower level of detection (LLD), a value was randomly selected from the tail of the distribution modelled using the whole cohort (>0 and below LLD). To account for seasonal differences in vitamin D status, reported 25OHD level was May-standardized by adjusting for sampling month using differences in age- and sex-adjusted monthly averages generated from SOCCS control data.[5] May-adjusted measurements were used in reported analyses, except in linear regression models, where unadjusted 25OHD level was used with month of sampling added as an additional variable, as previously described.[6]

Statistical analysis was conducted in R,[7] using the Survival, Hmisc, pROC and ROCR packages and charts derived using ggplot2.[8] In the CRC cohort analyses,

25OHD values were allocated to a time point category either preoperative or postoperative, with postoperative samples grouped by 12-monthly intervals. We used the paired Wilcoxon sign-ranked test to compare mean ranks of 25OHD at different perioperative timepoints. We explored the putative associations between clinical and demographic factors and perioperative changes in 25OHD using multivariable linear regression modelling. Spearman rank correlation and linear mixed-effects modelling from the 'lme4' R package were used for serial sampling study (i.e. repeated measures model) to assess association between perioperative changes in CRP and 25OHD.
Wilcoxon sign-ranked test was used to compare mean ranks of 25OHD in the large CRC cohorts by timepoint category and a multivariable linear regression model including factors with an established/putative impact on 25OHD (age, gender, BMI, vitamin D supplement use, AJCC and genetic score [9]) was performed to adjust for the association between these demographic and genetic factors on 25OHD. Survival analysis was performed using the 'survival' package in R using Cox proportional hazards models to calculate hazard ratios (HRs), adjusting for other relevant factors including CRP and time from surgery to sample date.
Test of the proportional hazards assumptions was performed and HRs were calculated for vitamin D tertiles, with the lowest category as the reference. The extracted HRs and 95%CIs were used to calculate the pooled HR estimates across CRC cohorts 1 and 2. The standard errors (SE) were used to calculate weighting for each study. Due to observed heterogeneity between the two cohorts we implemented the DerSimonian and Laird random-effects model to estimate pooled HR. All analyses were performed using the R-package ‘metafor’.[10] The main effect of a previously reported *VDR* polymorphism (rs11568820) [2] and its multiplicative interaction with vitamin D on survival was assessed by using a Cox proportional hazards model. Finally, the individual cohort survival models without and with vitamin D were compared using an ANOVA likelihood ratio test with AUC calculated in R. A logistic regression model was created for data truncated at 5-years to allow development of a clinical relevant calculator. For all analysis P-value <0.05 was considered statistically significant.

References

1. Knox S, Harris J, Calton L, et al. A simple automated solid-phase extraction procedure for measurement of 25-hydroxyvitamin D3 and D2 by liquid chromatography-tandem mass spectrometry. *Ann Clin Biochem* 2009;46(Pt 3):226-30. doi: 10.1258/acb.2009.008206
2. Zgaga L, Theodoratou E, Farrington SM, et al. Plasma vitamin D concentration influences survival outcome after a diagnosis of colorectal cancer. *J Clin Oncol* 2014;32(23):2430-9. doi: 10.1200/JCO.2013.54.5947
3. Kimlin MG, Lucas RM, Harrison SL, et al. The contributions of solar ultraviolet radiation exposure and other determinants to serum 25-hydroxyvitamin D concentrations in Australian adults: the AusD Study. *Am J Epidemiol* 2014;179(7):864-74. doi: kwt446 [pii]

10.1093/aje/kwt446 [published Online First: 2014/02/28]
4. Mithal A, Wahl DA, Bonjour JP, et al. Global vitamin D status and determinants of hypovitaminosis D. *Osteoporos Int* 2009;20(11):1807-20. doi: 10.1007/s00198-009-0954-6
5. Zgaga L, Theodoratou E, Farrington SM, et al. Diet, environmental factors, and lifestyle underlie the high prevalence of vitamin D deficiency in healthy adults in Scotland, and supplementation reduces the proportion that are severely deficient. *J Nutr* 2011;141(8):1535-42. doi: 10.3945/jn.111.140012
6. Freckleton R. On the misuse of residuals in ecology: regression of residuals vs. multiple regression. *Journal of Animal Ecology* 2002;71
7. R: A language and environment for statistical computing [program]: R Foundation for Statistical Computing, Vienna, Austria, 2013.
8. Wickham H. ggplot2: Elegant Graphics for Data Analysis: Springer Publishing Company, Incorporated 2009.
9. Jiang X, O'Reilly PF, Aschard H, et al. Genome-wide association study in 79,366 European-ancestry individuals informs the genetic architecture of 25-hydroxyvitamin D levels. *Nat Commun* 2018;9(1):260. doi: 10.1038/s41467-017-02662-2
10. Viechtbauer W. Conducting Meta-Analyses in R with the metafor Package. *Journal of Statistical Software* 2010;36(3):1-48.
