# Supplementary material for: Low plasma vitamin D is associated with adverse colorectal cancer survival after surgical resection, independent of systemic inflammatory response
Source: Gut. 2019 Apr 25;69(1):103–11. doi: 10.1136/gutjnl-2018-317922 (PMC6943245; doi:10.1136/gutjnl-2018-317922)
Supplement: Supplementary file 2 [file gutjnl-2018-317922supp002.pdf]

Supplementary Tables and Figures

Supplementary Table 1 Peri-operative CRP in serial sampling study

** Paired Wilcoxon sign rank test used to compare levels.*

Time-point (days)	N	Mean CRP mg/l (95%CI)	P*
**Preoperative**	70	13.5 (4.3-22.8)	reference
**Intraoperative**	30	11.7 (-2.3-25.77)	0.37
**1-2**	39	100.6 (84.5-116.8)	1.60e-14
**3-5**	38	143.1 (118.4-167.8)	1.39e-12
**6-9**	31	77.5 (58.3-96.7)	1.96e-6
**30-120**	40	15.4 (3.1-27.7)	0.51
**>162**	39	6.7 (3.4-10.0)	0.14

Supplementary Table 2 Linear mixed-effects regression model for serial sampling study for association between 25OHD and sample time point before and after adjustment for contemporaneous CRP level

*Linear mixed model including age, AJCC, gender and BMI fitted as fixed effects and patient as a random effect. *Model additionally adjusted for CRP with estimated contribution of CRP to the adjusted model given.*

Time point	*Unadjusted*		*Adjusted for CRP*	
	Estimate	P	Estimate*	P*
**Preoperative**	Reference	Reference	Reference	Reference
**Intraoperative**	-13.27	5.25e-05	-13.81	7.78e-5
**1-2 days**	-17.89	2.38e-13	-18.50	5.52e-7
**3-5 days**	-17.80	7.10e-13	-17.20	6.38e-5
**6-9 days**	-11.24	1.72e-05	-11.33	0.002
**30-120 days**	-6.90	0.004	-6.11	0.04
**>162 days**	17.48	3.04e-10	17.41	1.99e-8
**CRP**	NA	NA	0.01	0.69

Supplementary Table 3 Contribution of demographic and clinical factors to 25OHD level in Cohort 1 and Cohort 2

3354 patients with complete data for all variables examined in linear regression models. Raw 25OHD used (i.e. not adjusted for seasonal variation), with model adjusted for sample time-point and sample month. Data on chemotherapy use was available for a subset of patients in each cohort (n=314 in cohort 1, n=1452 in cohort 2) yet was not associated with 25OHD after adjustment for other factors and so not included in the model.

	Cohort 1		Cohort 2	
	Estimate	P	Estimate	P
Age	-0.28	2.2e-10	0.02	0.77
BMI	-0.45	1.7e-5	-0.65	3.8e-9
Gender (M)	-0.74	0.42	-1.72	0.15
AJCC	-1.28	0.008	-0.76	0.25
Vitamin D supplements (Y)	6.15	0.03	14.08	3.1e-16

Supplementary Table 4 May-adjusted 25OHD levels in Cohort 1 and Cohort 2 stratified by sample time point

*Difference in 25OHD assay in cohort 1 and cohort 2 precludes direct comparison between two cohorts. Time point categories given as months postoperatively; Mean May-adjusted 25OHD given as nmol/l with 95%CI; *linear regression model using raw 25OHD adjusting for age; gender; BMI; AJCC; vitamin D supplement use and month of sampling; p value reflects comparison with 0-12 months postoperative sampling values. Preoperative samples taken up to 154 days before surgery, postoperative samples taken up to 7624 days from surgery. Full results with effect size estimates stratified by 12-month blocks given in table below.*

Time point	N	CRC cohort 1 25OHD (nmol/l)	Est.*	P*	N	CRC cohort 2 25OHD (nmol/l)	Est.*	P*
**All**	**2006**				**2100**	**51.66 (50.51-52.81)**	**-**	**-**
**Preop.**	-	-	-	-	560	54.8 (52.4-57.1)	7.27	1.2e-5
**0-12**	1798	29.2 (28.3-30.1)	ref	ref	549	48.8 (46.6-51.0)	ref	ref
**12-24**	180	30.1 (27.0-33.2)	93	0.35	396	50.4 (47.9-52.8)	2.17	0.22
**>24**	28	37.8 (30.6-45.0)	2.54	0.56	595	52.2 (50.0-54.5)	4.81	0.002

Supplementary Table 5 Survival analysis for all cause death in Cohort 1 and Cohort 2 stratifie d by 6-monthly sample time-point

Model adjusted for age, sex, AJCC stage, body mass index, tumour site (colon/rectum), body mass index, tumour site (colon/rectum), time between definitive treatment and sampling. *Combined HR calculated by meta-analysis of Cohorts 1 and 2.*

	N	Tertile 1	Tertile 3		
Cohort 1		**HR**	**HR**	**95% CI**	**P**
0-6 months	1058	ref	0.74	0.58-0.94	0.01
6-12 months	286	ref	0.56	0.36-0.86	0.01
12-18 months	97	ref	0.64	0.30-1.37	0.24
18-24 months	29	ref	1.25	0.1-13.38	0.85
>24 months	16	ref	0.14	0.001-3.38	0.49
All patients	**1486**	**ref**	0.69	0.56-0.84	0.0002
Cohort 2		**HR**	**HR**	**95% CI**	**P**
Preop.	464	ref	0.30	0.12-0.71	0.006
0-6 months	247	ref	0.57	0.21-1.56	0.27
6-12 months	203	ref	0.99	0.28-3.42	0.98
12-18 months	172	ref	0.93	0.40-2.15	0.86
18-24 months	171	ref	0.64	0.28-8.71	0.62
>24 months	535	ref	0.66	0.36-1.20	0.17
All patients	1792	ref	0.63	0.44-0.89	0.01
Meta-analysis		**HR**			
0-6 months	1305	ref	0.73	0.50-0.96	-
6-12 months	489	ref	0.61	0.20-1.01	-
12-18 months	269	ref	0.77	0.21-1.33	-

18-24 months	199	ref	0.97	-0.78-2.73	-
>24 months	551	ref	0.64	0.05-1.23	-

Supplementary Table 6 Survival analysis in Cohort 2 Preoperative patients adjusted for CRP

	N	Tertile 1 *<38.0nmol/l*	Tertile 2 *38.0-57.9nmol/l*			Tertile 3 *>57.9nmol/l*		
**Model**		**HR**	**HR**	**95% CI**	**P**	**HR**	**95% CI**	**P**
CRC death								
**unadjusted**	486	ref	0.68	0.29-1.62	0.39	0.40	0.14-1.13	0.08
**model 2 ***	460	ref	0.69	0.28-1.67	0.41	0.43	0.15-1.30	0.14
**model 3 †**	**307**	**ref**	**0.91**	**0.33-2.46**	**0.86**	**0.73**	**0.24-2.24**	**0.58**
All-cause death								
**unadjusted**	485	ref	0.71	0.39-1.32	0.28	0.33	0.15-0.74	0.24
**model 2 ***	464	ref	0.70	0.37-1.30	0.26	0.30	0.12-0.71	0.006
**model 3 †**	**307**	**ref**	**0.79**	**0.39-1.60**	**0.51**	**0.38**	**0.15-0.98**	**0.04**

*NOTE. Final model is shown in bold. Abbreviations: 25OHD, 25-hydroxyvitamin D; CRC, colorectal cancer; HR, hazard ratio; Ref, reference. *model adjusted for age, sex, AJCC stage, body mass index, tumour site (colon/rectum), time between definitive treatment and sampling. Where data were missing, participants were excluded from adjusted model. †Multivariate model additionally adjusted for CRP. Only 23 CRC deaths in patients included in Model 3 may contribute to lack of statistical significance seen.*

Supplementary Table 7 Survival analysis in Cohort 1 and Cohort 2 stratified by May-adjusted 25OHD level cut-off
A recursive approach was taken to identify putative thresholds associated with overall survival and meta-analysis performed for those thresholds most strongly associated with survival.

**Cohort 1**	**HR**	**P**	**N above**
**25OHD>10 nmol/l**	0.72	0.002	1452 (86%)
**25OHD>15 nmol/l**	0.73	0.0003	1249 (74%)
**25OHD>20 nmol/l**	0.75	0.0003	1062 (63%)
**25OHD>25 nmol/l**	0.80	0.006	856 (51%)
**25OHD>30 nmol/l**	0.80	0.008	682 (40%)
**25OHD>35 nmol/l**	0.76	0.004	525 (31%)
**25OHD>40 nmol/l**	0.74	0.004	402 (24%)
**25OHD>45 nmol/l**	0.71	0.004	301 (18%)
**25OHD>50 nmol/l**	0.76	0.05	220 (13%)
**25OHD>55 nmol/l**	0.84	0.22	164 (10%)
**Cohort 2**	**HR**	**P**	**N above**
**25OHD>10 nmol/l**	1.11	0.89	1823 (99%)
**25OHD>15 nmol/l**	0.74	0.45	1794 (97%)
**25OHD>20 nmol/l**	1.07	0.82	1738 (94%)

**25OHD>25 nmol/l**	0.80	0.29	1647 (89%)
**25OHD>30 nmol/l**	0.70	0.03	1512 (82%)
**25OHD>35 nmol/l**	0.77	0.08	1342 (73%)
**25OHD>40 nmol/l**	0.72	0.02	1156 (63%)
**25OHD>45 nmol/l**	0.80	0.11	968 (52%)
**25OHD>50 nmol/l**	0.67	0.008	792 (43%)
**25OHD>55 nmol/l**	0.69	0.02	682 (37%)
**25OHD>60 nmol/l**	0.65	0.01	569 (31%)
**25OHD>65 nmol/l**	0.78	0.16	477 (26%)
**25OHD>70 nmol/l**	0.83	0.34	384 (21%)
**Meta-analysis**	**HR**	**95%CI**	**-**
**25OHD>45 nmol/l**	0.75	0.57-0.93	-
**25OHD>50 nmol/l**	0.72	0.52-0.92	-

Supplementary Figure 1 Meta-analysis of 25OHD tertile 3 and tertile 1 with CRC-specific and all-cause mortality adjusted for CRP
Adjusted hazard ratios used for meta-analysis, adjusted for CRP, age, sex, AJCC stage, body mass index, tumour site (colon/rectum) and time between definitive treatment and sampling.

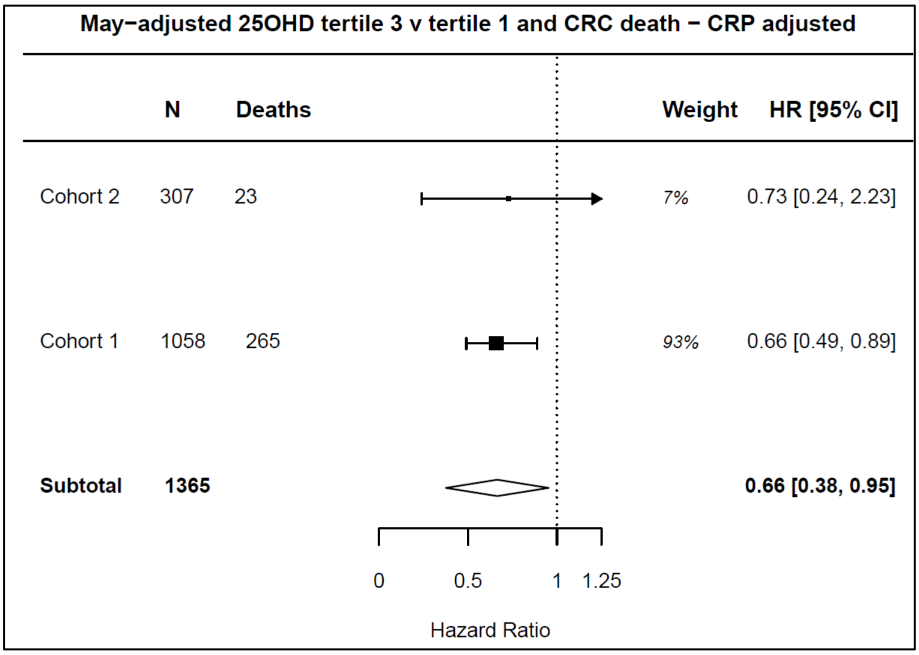

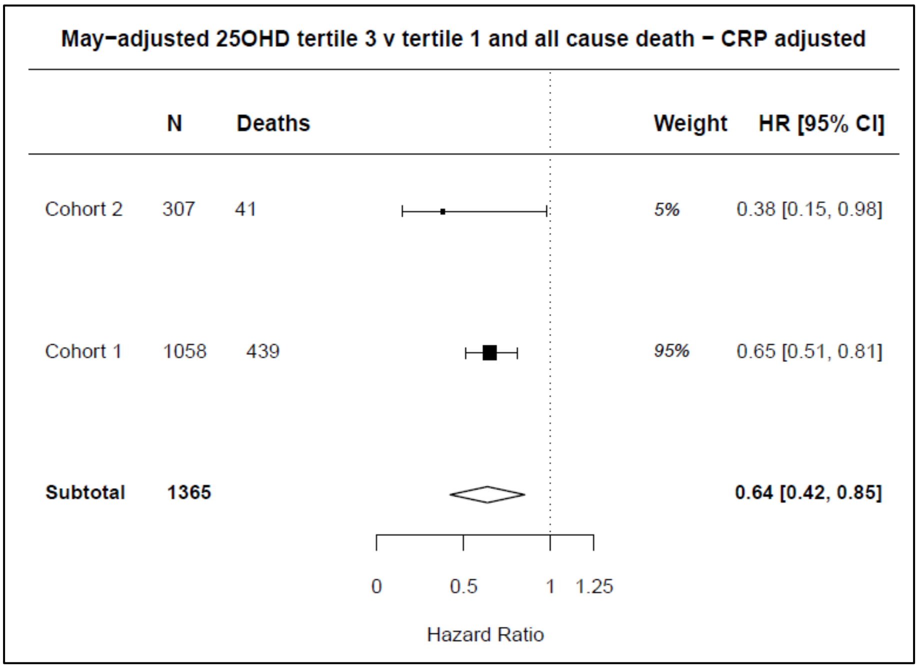

Supplementary Table 8 Difference in survival between Cohort 1 and Cohort 2
Adjusted for age, gender, BMI, site, AJCC, sample time from surgery (< >12 months)

	**Cohort 1** *Mean 25OHD 29.17*	**Cohort 2** *Mean 25OHD 46.89*		
**Model**	**HR**	**HR**	**95% CI**	**P**
CRC death	ref	0.58	0.42-0.79	0.0005
All-cause death	ref	0.63	0.49-0.80	0.0002

Supplementary Table 9 Multivariable-adjusted analysis of HR of CRC mortality in Cohort 1 and Cohort 2 according to polymorphisms in VDR gene and their interaction with may-adjusted plasma 25OHD concentration
Multivariate model adjusted for age, gender, AJCC, cancer site and BMI .For CRC Cohort 1 model was repeated with addition of CRP with the interaction term for rs11568820 P = 0.003 (n=1018 cases included). For CRC Cohort 2 only 282 cases with full genotyping and CRP level were available and so CRP was not included as a covariate. No significant interaction seen for all cause death in Cohort 2, interaction P=0.002 for all cause death in Cohort 1.

		**Cohort 1**			**Cohort 2**		
**SNP**	**Model**	**SNP**	**25OHD**	**$P_{interaction}$**	**SNP**	**25OHD**	**$P_{interaction}$**
**rs11568820** (AA or AG *v* GG†)	Simple	0.64	0.03	-	0.62	0.14	-
	With interaction	0.12	0.002	**0.02**	0.04	0.02	**0.03**

Supplementary Table 10 Survival analysis in Cohort 1 and Cohort 2 stratified by rs11568820 genotype
Multivariate model adjusted for age, gender, AJCC, cancer site, BMI and time to sampling.

		**Tertile 1** *<38.0nmol/l*	**Tertile 2** *38.0-57.9nmol/l*			**Tertile 3** *>57.9nmol/l*			
**CRC death**	**N**	**HR**	**HR**	**95% CI**	**P**	**HR**	**95% CI**	**P**	**P trend**
Cohort 1									
rs11568820 AA/AG	546	ref	1.36	0.89-2.07	0.15	1.11	0.72-1.71	0.65	0.49
rs11568820 GG	871	ref	0.69	0.50-0.94	0.02	0.54	0.38-0.77	5.7e-4	6.4e-4
Cohort 2									
rs11568820 AA/AG	620	ref	0.84	0.40-1.77	0.64	1.03	0.51-2.10	0.93	0.76
rs11568820 GG	1090	ref	0.67	0.41-1.09	0.11	0.43	0.24-0.77	0.005	0.015
Meta-analysis									
rs11568820 AA/AG	1166	ref	1.19	0.72-1.67		1.09	0.72-1.46	-	-
rs11568820 GG	1961	ref	0.68	0.42-0.95		0.51	0.21-0.81		
**All-cause death**	**N**	**HR**	**HR**	**95% CI**	**P**	**HR**	**95% CI**	**P**	**P trend**
Cohort 1									
rs11568820 AA/AG	546	ref	0.91	0.67-1.26	0.58	0.91	0.62-1.25	0.56	0.83
rs11568820 GG	871	ref	0.69	0.54-0.87	0.002	0.58	0.45-0.76	4.8e-5	1.5e-5
Cohort 2									
rs11568820 AA/AG	620	ref	1.12	0.64-1.97	0.69	0.87	0.48-1.56	0.64	0.89
rs11568820 GG	1090	ref	0.72	0.48-1.09	0.12	0.52	0.32-0.84	0.007	0.03
Meta-analysis									
rs11568820 AA/AG	1136	ref	0.96	0.68-1.24		0.90	0.62-1.18	-	-
rs11568820 GG	1934	ref	**0.70**	0.49-0.90		0.57	0.33-0.80	-	-

Supplementary Table 11 Results of multivariate logistic regression model for all-cause mortality (Yes/ No) in Cohort 1 and Cohort 2 at 5 years and raw 25OHD

**Cohort 1**	**Odds ratio**	**P value**
25OHD (per 1 nmol/l increment)	0.99	0.02
SampleMonth2	0.80	0.48
SampleMonth3	0.67	0.23
SampleMonth4	0.62	0.16
SampleMonth5	1.62	0.12
SampleMonth6	0.97	0.93
SampleMonth7	1.04	0.90
SampleMonth8	0.78	0.47
SampleMonth9	0.79	0.51
SampleMonth10	0.77	0.42
SampleMonth11	0.92	0.78
SampleMonth12	0.79	0.49
Age	1.02	0.01
Sex (M)	1.22	0.14
AJCC	2.67	2.9e-24
Sampling less than 12 months	0.96	0.87
CRC Site (Rectum)	1.44	0.01
**Cohort 2**		
25OHD (per 1 nmol/l increment)	0.99	0.015
SampleMonth2	0.30	0.03
SampleMonth3	0.70	0.41
SampleMonth4	0.53	0.18
SampleMonth5	0.76	0.56
SampleMonth6	0.92	0.86
SampleMonth7	0.79	0.66
SampleMonth8	1.15	0.77
SampleMonth9	1.15	0.77
SampleMonth10	0.77	0.60
SampleMonth11	0.64	0.35
SampleMonth12	0.69	0.53
Age	1.04	2.14e-04
Sex (M)	1.21	0.38
AJCC	2.35	1.89e-08
Sampling less than 12 months	9.12	4.33e-11
CRC Site (Rectum)	1.35	0.18

Supplementary Table 12 AUC for survival model for all-cause mortality at 5 years without and with raw 25OHD
**P value calculated using ANOVA likelihood ratio test of difference between two logistic regression models. Sample time reflects sample time from surgery (< >12 months)*

Cohort	N	Model 1<br><br>AJCC + sample time + sample month	Model 2<br><br>AJCC, age, gender, BMI, cancer site +sample time + sample month	Model 3<br><br>All clinical variables + 25OHD + sample month	P value* Model 1 *vs* Model 3	P value* Model 2 *vs* Model 3
CRC Cohort 1	1687	0.68	0.718	0.723	5.9e-8	0.04
CRC Cohort 2	585	0.71	0.74	0.77	0.0002	0.01
